# A Randomized Controlled Trial on the Effects of Aerobic and Coordinative Training on Neural Correlates of Inhibitory Control in Children

**DOI:** 10.3390/jcm8020184

**Published:** 2019-02-04

**Authors:** Sebastian Ludyga, Flora Koutsandréou, Eva-Maria Reuter, Claudia Voelcker-Rehage, Henning Budde

**Affiliations:** 1 Department of Sport, Exercise and Health, University of Basel, 4052 Basel, Switzerland; 2 Faculty of Human Sciences, Medical School Hamburg, 20457 Hamburg, Germany; florakouts@gmail.com (F.K.), henning.budde@medicalschool-hamburg.de (H.B.); 3 Centre for Sensorimotor Performance, School of Human Movement and Nutrition Sciences, The University of Queensland, Brisbane, QLD 4072, Australia; e.reuter@uq.edu.au; 4 Institute of Human Movement Science and Health, Faculty of Behavioral and Social Sciences, Chemnitz University of Technology, 09111 Chemnitz, Germany; claudia.voelcker-rehage@hsw.tu-chemnitz.de; 5 Institute of Sport Science and Innovations, Lithuanian Sports University, 44221 Kaunas, Lithuania; 6 Physical Activity, Physical Education, Health and Sport Research Centre (PAPESH), Sports Science Department, School of Science and Engineering, Reykjavik University, IS-101 Reykjavik, Iceland

**Keywords:** executive function, physical activity, aerobic fitness, motor skill, P300, event-related potentials, Flanker task

## Abstract

Whereas aerobic training has found to be beneficial for inhibitory control, less is known on the efficiency of other exercise types in children. The present study compared the effects of aerobic and coordinative training on behavioral and neurophysiological measures of inhibitory control. Forty-five children were randomly assigned (1:1:1 ratio) to groups performing aerobic training, coordinative training or assisted homework sessions over 10 weeks. Before and after intervention, all participants completed a Flanker task. The P300 component of event-related potentials elicited from the task was recorded via electroencephalography. Additionally, aerobic fitness and gross-motor skills were assessed using 20 m Shuttle Run and Heidelberg Gross-Motor Test, respectively. Statistical analyses revealed no time by group interactions for the P300 component (amplitude, latency), *p* = 0.976, η^2^ = 0.007, and behavioral performance (reaction time, accuracy), *p* = 0.570, η^2^ = 0.045. In contrast, there was a significant group-difference in pre- to post-test changes in aerobic fitness, *p* = 0.008, η^2^ = 0.246, with greater improvements following aerobic and coordinative training compared to assisted homework sessions. In conclusion, no differences regarding the efficiency of aerobic and coordinative training for the enhancement of inhibitory control were found as both exercise programs failed to elicit changes in speed and accuracy of stimulus evaluation and the allocation of attentional resources.

## 1. Introduction

The benefits of regular exercise for aerobic fitness and motor skills appear to be well-documented and evidence suggests that these may also span to other domains relevant for children’s health [1,2,3]. Based on the association between different aspects of physical fitness and cognitive performance [4], the interest in physical training programs as potential strategy to elicit cognitive enhancements has been growing. Given the need to differentiate training from physical activity [5], it is defined as planned, structured, and repetitive process with the aim of improving and maintaining physical fitness [6]. In addition, executive functions have been rendered an important target for such training interventions [7]. These functions can be understood as a set of abilities needed for top-down control of behavior, especially in less familiar or novel situations. From an educational perspective, executive functions are highly relevant due to their association with school readiness [8] and academic achievement, including both math and literacy skills [9]. This can partly be explained by the predictive role of the inhibitory aspect of executive functioning (i.e., the ability to suppress unwanted thoughts, feelings or actions) for competences crucial in learning [10], such as self-regulation [11] and social-emotional competences [12].

Children’s executive functions seem to be sensitive to regular exercise as meta-analytical findings support small, but significant improvements in this cognitive domain following a period of regular engagement in exercise [13]. With regard to inhibitory control, subgroup-analysis further revealed that exercise programs challenging cognitive functions and/or body coordination to greater extent were most beneficial. However, it should be noted that this conclusion was drawn from comparisons across rather than within studies. In this respect, previous randomized-controlled trials reported enhancements of children and adolescents’ inhibitory control after a period of mainly aerobic training [14,15], exercise with additional coordinative or cognitive demands [16,17] and a combination of both [18,19]. As there is a paucity of studies that directly compare the cognitive benefits of different exercise programs, evidence on their efficiency for enhancing the inhibitory aspect of executive function is still limited.

Given that aerobic fitness [20] and qualitative aspects of motor skills [21] are both linked to inhibitory control, training programs that aim for improvements in this outcome have the potential to elicit enhancements in this cognitive domain. Although this might explain the cognitive benefits of aerobic and coordinative training, it remains unclear if these are triggered by the same pathway. In general, the mechanisms underlying physical training-induced improvements in different cognitive domains are still a matter of debate [22]. Using electroencephalography and by applying the event-related potential (ERP) technique, effects of aerobic and coordinative training can be studied on a neurocognitive level, so that insights into the cognitive process by which exercise influences task performance can be gained. The P300 component of ERPs is a candidate that allows for the investigation of inhibition mechanisms in tasks that tap the inhibitory aspect of executive function [23]. The latency of this component is suggested to reflect stimulus evaluation speed, whereas its amplitude indexes the allocation of attentional resources and the inhibition of superfluous neural activity to meet the demands in the transmission of stimulus information [24]. 

A review focusing on the association between aerobic and specific ERPs suggests that high-fit children show shorter P300 latency and higher P300 amplitude than their less-fit peers, especially on cognitive tasks that demand high inhibitory control [25]. This can be understood as a greater availability of attentional resources, which are allocated to quickly process relevant stimulus information despite high task demands, in children with high aerobic fitness. These cross-sectional findings have been supported by results from randomized controlled trails (RCTs), although it should be noted that the effects of physical training on the P300 component elicited from an inhibitory control task have rarely been investigated in children [26]. Following 9 months of training with the primary aim to enhance aerobic fitness, Hillman et al. [14] have reported a greater increase in P300 amplitude and decrease in P300 latency on trials demanding high inhibitory control in the exercise compared to the control group. Similar effects on amplitude and latency of the P300 component were observed after a much shorter intervention period in kindergarten children, who engaged in an 8-week coordinative training program [27]. In both studies, changes in the P300 amplitude were paralleled by improvements on task performance. However, it should be noted that the study employing coordinative training was lacking a control group. In contrast to the sole examination of specific training modalities, Ludyga et al. [19] have investigated the effects of combined aerobic and coordinative training on young adolescents’ inhibitory control on both behavioral and neurocognitive levels. Over the 8-week intervention period, children in the exercise compared to the control group showed a greater increase in P300 amplitude, which was significantly correlated with a decrease in reaction time. In sum, this limited evidence suggests that an increased allocation of attentional resources is linked with an improved behavioral performance on inhibitory control tasks and that such changes can occur even after a short-term training intervention. Due to the small number of RCTs in this field and the lack of comparisons between the effects of aerobic and coordinative training within studies, it remains unclear if both training modalities influence the P300 component to the same degree in children. However, such insights can provide first indications on whether or not aerobic and coordinative training elicit improvements in inhibitory control via similar underlying mechanisms.

The purpose of the present study was the comparison of the effects of aerobic and coordinative training on inhibitory control and the P300 component of ERPs in children. In comparison to the control group, children allocated to the training groups were expected to show a greater increase of P300 amplitude and/or decrease of latency as well as greater improvements on task performance [14,19,27]. Based on meta-analytical findings [13], it was further assumed that these changes were of greater magnitude following coordinative training, when compared to the effects of aerobic training.

## 2. Materials and Methods

### 2.1. Participants

As recommended by Faul et al. [28], a power analysis was conducted a priori using G*Power 3.1. (Heinrich Heine University Düsseldorf, Düsseldorf, Germany). Previous studies investigating the effects of aerobic training, coordinative training or a combination of both on inhibitory control and P300 amplitude have reported small to large effect sizes [14,19,27]. Due to this heterogeneity, power analysis was based on meta-analytical findings showing a moderate effect (d = 0.49) of cognitively-demanding/coordinative training on children’s behavioral performance on an inhibitory control task [13]. Given an alpha level of *p* = 0.05 for testing the within-between subjects interaction, 39 participants were required to reach a power of 85%. As drop-outs were expected, the sample was increased by 2 participants per group.

The present study included a subsample (female: *N* = 20; male: *N* = 25) of a RCT [29]. This subsample was randomly selected and completed additional assessments (EEG recordings and a modified Flanker task). All participants were recruited from local schools and inclusion criteria were 9–10 years of age, right-handedness, corrected-to or normal vision and prepubescent status according to parent and self-report on the Tanner staging system [30,31]. In case of the presence of mental and physical impairments and/ or previous or actual intake of psychoactive substances, participants were deemed ineligible. Before the study commenced, the ethics committee of the German Psychological Society approved the protocol (HB 02201 6_amd_092011). All participants and their legal guardians provided informed written consent after study procedures were explained in detail. The study was conducted following the guidelines set forth in the declaration of Helsinki and registered in the German Clinical Trials Register (DRKS00016590).

### 2.2. Design

Participants were randomly assigned to an aerobic training group (AER; *N* = 15), a coordinative training group (COR; *N* = 15) and a control group (CON; *N* = 15). The intervention period in AER and COR lasted 10 weeks and was not interrupted by any school holidays. At pre- and post-test, all participants completed a computer-based Flanker task, the 20 m Shuttle-Run and the Heidelberg Gross-Motor Test (HGMT) [32]. During the Flanker task, stimulus-locked event-related potentials were recorded using electroencephalography. Over the data collection period, outcome assessors were blinded to the participants’ group allocation.

### 2.3. Training Intervention

Over the intervention period, the amount of physical education lessons was identical between groups (3 × 45 min per week). In addition, both intervention groups completed a training program, which consisted of three sessions of 45 min duration per week, during leisure time (after school). An instructor experienced in aerobic and coordinative training with children supervised these sessions. The aerobic training program in AER aimed to improve cardiovascular fitness and included age-appropriate running-based games at moderate-to-vigorous intensity, but low coordinative demand. In contrast, the goal of the training program in COR was the elicitation of benefits for fine and gross motor body coordination. Each session consisted of playful exercises, which placed demands on balance, bilateral coordination as well as spatial orientation and reaction to moving objects/persons. Some examples for the exercise sessions prescribed in AER and COR are provided in the Supplementary. In both intervention groups, the structure of the training and applied exercise types remained unchanged over the intervention period. Additionally, there was no progression towards higher intensity and/or complexity in AER and COR.

In a previous review on training programs that aim to improve cognition, passive control groups have been criticized as these may result in differences to the intervention group (e.g. motivation, expectations, engagement and interaction with the experimenter) that confound the results [33]. To reduce the risk that attention bias confounds the results, participants in CON took part in assisted homework sessions. During all sessions, a supervisor supported participants in finding a way to solve their homework. Problems and possible solutions of specific tasks were openly discussed with all participants. This approach was chosen to have a level of social interaction that is comparable to the one in AER and COR. The structure of the assisted homework sessions remained the same throughout the study and the participants’ progress was not monitored. The frequency and duration of these sessions was equal to those of AER and COR.

In all groups, participants’ heart rates were recorded continuously during three sessions (in weeks 2, 6 and 10) over the intervention period using F1 heart rate monitors (Polar Oy, Kempele, Finland). For statistical analysis, the recorded heart rate was averaged over the entire session and the three recorded time points.

### 2.4. Cognitive Task

All participants completed a modified, computer-based version of the Flanker task [34], which was administered with Presentation software 17.0 (Neurobehavioral Systems, Berkeley, USA, CA). One central and four surrounding colored discs were employed as visual stimuli. The central and flanking discs appeared in the same color on congruent trials and in the opposite color on incongruent trials. In both conditions, the visual stimuli were presented either in red or green. On neutral trials, the flanking discs appeared in blue color. During the task, participants were required to press a button corresponding to the color of the central (target) disc. Each trial started with a fixation cross (300 ms), which was followed by a blank screen (200 ms). Subsequently, visual stimuli were presented focally for 200 ms and responses were allowed within a 1200 ms time-window. The inter-trial interval varied randomly between 800 and 1100 ms. Following a practice round with 20 trials, 3 blocks with 100 trials each were administered. The different trial types appeared with equal probability and their order was randomized. The median reaction time from response-correct trials and accuracy were calculated separately for the congruent and incongruent conditions and extracted for statistical analyses.

### 2.5. EEG Recording and Processing

A 32-channel active electrode system (ActiveTwo, BioSemi, Amsterdam, The Netherlands) was used to record EEG data. The electrodes were mounted into a nylon mesh cap and arranged according to the 10–20 system [35]. Six additional facial electrodes were placed to record vertical and horizontal eye movements (from electrodes placed above and at the outer canthi of the eyes) as well as mastoid potentials. The signal was band pass filtered (0.16–100 Hz) online and collected with a sampling rate of 2048 Hz. Prior to the recordings, the scalp electrode impedance was checked and reduced below 10 KΩ to assure good signal quality. Offline processing was performed with Brain Vision Analyzer 2.1 (Brain Products, Munich, Germany). EEG data was down-sampled to 512 Hz, re-referenced to average mastoids and submitted to high- (0.3 Hz) and low-pass filters (30 Hz). Ocular artifacts were detected from VEOG and HEOG channels and corrected by applying a regression-based approach [36]. From response-correct trials, equal-sized segments were built for the latency range from 100 ms before to 800 ms after stimulus onset. The resulting segments were baseline-corrected using the 100 ms prestimulus period and further inspected for artefacts by applying a threshold-based approach. Segments with voltage differences of less than 85 µV within 100 ms were averaged separately for the congruent and incongruent condition of the Flanker task. In contrast, neutral trials were disregarded, because the analysis of this condition was not required to answer the research question of the present study.

Based on previous literature [14,24,27] and own experience, the analysis of the P300 component of ERPs was performed within the 300–600 ms time-window. As the P300 elicited from the Flanker task shows a centroparietal distribution [23], waveforms from CP1, CP2, Cz, and Pz were pooled to build a region of interest. The P300 latency corresponded to the peak within the pre-specified latency range. Subsequently, the adaptive mean was calculated as the average amplitude over the period from 50 ms prior to and 50 ms after the peak [14].

### 2.6. Statistical Analyses

SPSS 25.0 (IBM, Armonk, NY, USA,) was employed for statistical analyses of the collected data. In advance, the Shapiro-Wilk test and the Levene test were applied to check for Gaussian distribution of the data and homogeneity of variances, respectively. Possible group-differences in pre-test values (anthropometric data, fitness, motor coordination and main outcomes) and completed training/assisted homework sessions were assessed using one-way analyses of variance (ANOVAs). As manipulation check, heart rate was compared between groups using a series of paired *t*-tests. Additionally, a 2 (time: pre-test, post-test) × 3 (group: AER, COR, CON) ANOVA was applied separately on completed stages on the Shuttle Run and total score on the HGMT.

The effects of the training interventions on the P300 amplitude and latency were examined by applying a 2 (time) × 2 (congruency: congruent trials, incongruent trials) × 3 (group) multivariate analysis of variance (MANOVA). For the examination of behavioral performance, a similar MANOVA was conducted with reaction time and accuracy included as the dependent variables. In an additional analysis, gender was included as between-subjects factor (see Supplementary). In case of main effects and/or interactions, these were further examined using univariate analyses. Significant interactions were decomposed using a series of Bonferroni-corrected *t*-Tests. For all statistical comparisons, the level of significance was set to *p* < 0.05.

## 3. Results

Pre- and post-tests were completed by 36 participants, so that there were 8 drop-outs (AER: *N* = 4; COR: *N* = 3, CON: *N* = 1). These drop-outs were due to illness (*N* = 3) and personal reasons (*N* = 5). The characteristics of the remaining participants are shown in Table 1. Additionally, ERP data from one participant in COR could not be analyzed as it was contaminated by artefacts. The comparison of anthropometric measures, completed stages on the Shuttle Run, score on the HGMT, performance on the Flanker task (reaction time and accuracy) as well as amplitude and latency measures of the P300 at pre-test revealed no significant differences between groups. Moreover, there was no difference in the number of completed training/ assisted homework sessions (of 29 scheduled sessions) between AER (*M* = 27.9, *SD* = 1.2), COR (*M* = 26.9, *SD* = 1.4) and CON (*M* = 26.9, *SD* = 1.7), *F*(2,34) = 1.67, *p =* 0.203, η^2^ = 0.090.

### 3.1. Heart Rate, Aerobic Fitness and Gross-Motor Skills

AER (*M* = 140.3 bpm, *SD* = 10.5) showed a higher heart rate compared to COR (*M* = 124.4 bpm, *SD* = 12.3), *T*(21) = 3.29, *p* = 0.003. Moreover, both groups had a higher heart rate during training than CON during assisted homework sessions (*M* = 79.4 bpm, *SD* = 6.0), *T*(24) ≥ 12.09, *p* ≤ 0.001.

With regard to aerobic fitness and gross motor skills, there was a time by group interaction for completed stages on the Shuttle Run, *F*(1,34) = 5.53, *p* = 0.008, η^2^ = 0.246, whereas no such interaction was found for total score on the HGMT. Both AER, *T*(10) = 4.47, *p* = 0.001, and COR, *T*(11) = 2.58, *p* = 0.026, completed more stages at post- compared to pre-test, whereas there was no change in CON (Table 2).

### 3.2. Behavioral Performance

The MANOVA revealed a significant main effect of time, Wilks’λ = 0.501, *F*(2,33) = 16.13, *p <* 0.001, η^2^ = 0.494, and congruency, Wilks’λ = 0.212, *F*(2,33) = 61.24, *p* < 0.001, η^2^ = 0.788, on cognitive performance (Table 3). Further examination using univariate analyses showed an increase of accuracy from pre-test(*M* = 70.2%, *SD* = 13.3) to post-test (*M* = 79.4 %, *SD* = 11.5) across groups, *F*(1,34) = 31.39, *p* < 0.001, η^2^ = 0.480. Moreover, participants had lower reaction time, *F*(1,34) = 96.52, *p* < 0.001, η^2^ = 0.740, and higher accuracy, *F*(1,34) = 34.18, *p* < 0.001, η^2^ = 0.501, on congruent (reaction time: *M* = 705.3 ms, *SD* = 117.7; accuracy: *M* = 77.6%, *SD* = 11.3) compared to incongruent trials (reaction time: *M* = 749.9 ms, *SD* = 128.3; accuracy: *M* = 72.0%, *SD* = 12.3) across groups. No time x group interaction, Wilks’λ = 0.986, *F*(4,66) = 0.12, *p* = 0.976, η^2^ = 0.007, and time x group x congruency interaction were found, Wilks’λ = 0.967, *F(4*,66) = 0.89, *p* = 0.112, η^2^ = 0.124.

### 3.3. P300 Component

**Figure 1:**
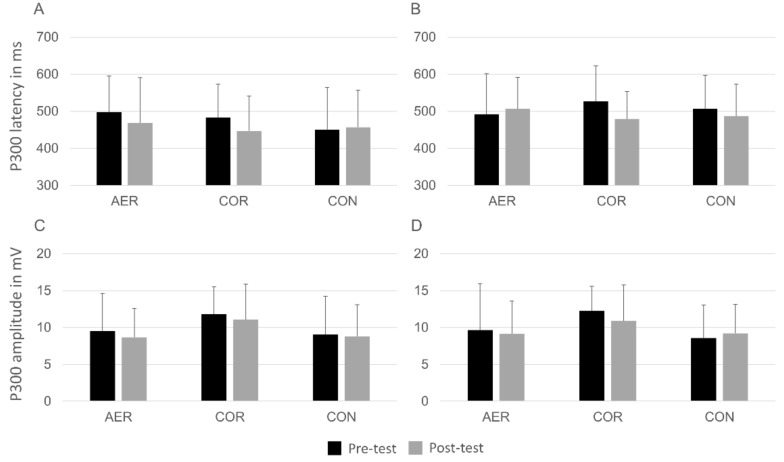
Means and standard deviation for P300 latency ((**A**) congruent trials; (**B**) incongruent trials) and amplitude ((**C**) congruent trials; (**D**) incongruent trials) in all groups at pre and post-test. Note: AER = aerobic training group; COR = coordinative training group; CON = control group.

**Figure 2:**
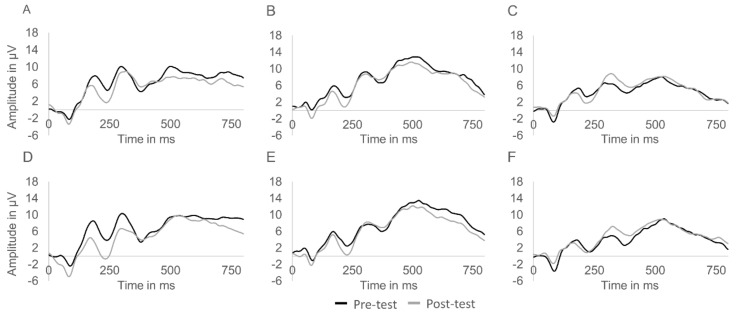
Centroparietal event-related potential waveforms of the aerobic ((**A**) congruent trials; (**D**) incongruent trials) and coordinative training ((**B**) congruent trials; (**E**) incongruent trials) groups as well as the control group ((**C**) congruent trials; (**F**) incongruent trials) at pre- and post-test. Note: Waveforms were pooled from Cz, Pz, Cp1, and Cp2.

With regard to the P300 component, there was a multivariate main effect of congruency, Wilks’λ = 0.784, *F*(2,31) = 4.27, *p* = 0.023, η^2^ = 0.216. The examination of this effect using univariate analyses revealed that P300 latency was shorter on congruent (*M* = 466.8 ms, *SD* = 93.3) compared to incongruent trials (*M* = 499.1 ms, *SD* = 80.9), *F*(1,32) = 8.87, *p =* 0.006, η^2^ = 0.215. No time × group interaction, Wilks’λ = 0.911, *F*(4,62) = 0.74, *p* = 0.570, η^2^ = 0.045, and time × group × congruency interaction were found, Wilks’λ = 0.881, *F(4*,62) = 1.02, *p* = 0.405, η^2^ = 0.062. (Figure 1). The event-related potential waveforms of all groups at pre- and post-test are displayed in Figure 2.

## 4. Discussion

The present study addressed the research deficit regarding the effects of the exercise modality on behavioral and neurophysiological indices of inhibitory control by comparing aerobic and coordinative training. Despite a high training compliance as indicated by participants completing more than 90% of the scheduled sessions, pre- to post-test changes in P300 amplitude and latency were not different between groups. With regard to behavioral performance, there was an increase of accuracy over the intervention period, but similar to the ERP findings, the magnitude of changes did not differ between groups.

The absence of improvements in reaction time and accuracy beyond learning effects following both aerobic and coordinative training is in conflict with previous studies showing physical training-induced benefits for inhibitory control in children [14,15,16,17,18,19]. Although the sensitivity of this core component of executive function to different exercise modalities appears to be consistent [13], a few studies have obtained a pattern of results similar to those of the present study. For example, Schmidt et al. [37] did not observe improvements in inhibitory control following 6 weeks of aerobic training (with either low or high cognitive demands). A lack of benefits for this executive function component was also reported after a much longer intervention program, which aimed to foster moderate-to-vigorous physical activity within the school curriculum [38]. Similar to the present findings, the training interventions in both studies led to an increase in children’s aerobic fitness, whereas their inhibitory control remained unchanged. This is surprising, given that a review supports an association between aerobic fitness and executive function [20]. Additionally, evidence from cross-sectional and experimental studies suggests that some facets of motor coordination are linked with inhibitory control [16,21]. As the coordinative training failed to elicit improvements in gross-motor skills assessed with the HGMT, this may offer a possible explanation for the lack of inhibitory control benefits. This lack of effects is in conflict with meta-analytical findings showing that independent of the length of the intervention, coordinative training elicits small-to-moderate effects on such motor skills in children [39]. However, it should be noted that there was a tendency towards higher gross-motor skills in COR compared to AER and CON at pre-test, so that ceiling effects may have affected the efficiency of the training. Moreover, the complex nature of the tasks included in coordinative training required a longer instruction time than in the aerobic training program, thus reducing valuable movement time. Consequently, extending the time spent performing coordinative training might have been necessary to elicit the improvements in motor skills the program aimed for.

With regard to the P300 component, the pattern of results was similar to behavioral findings. Neither amplitude, nor latency measures of the P300 were influenced by aerobic or coordinative training. Both speed of stimulus classification and the allocation of attentional resources are processes that contribute to inhibitory control [23], but do not alone determine behavioral performance. Therefore, it is possible that changes in these processes are evidenced by a facilitation of P300 amplitude and latency, even before a beneficial effect on reaction time and accuracy is observable. The present findings provide no indication for a training-induced alteration of the P300 component, so it seems unlikely that an extended intervention period would have resulted in beneficial effects on inhibitory control. Whereas a previous study has reported increased P300 amplitude and decreased P300 latency after a much longer intervention (9 months) [14], others observed similar effects after a comparable duration and even lower time spent with performing the scheduled training sessions [19,27]. Since the positive association between aerobic fitness and P300 amplitude is well-documented in children [25], it is questionable why the improvements of aerobic fitness found in both AER and COR have not been accompanied by a favorable alteration in the allocation of attentional resources. Based on normative values for aerobic fitness assessed from the Shuttle Run [40], the sample in the present study roughly corresponded to the 70th percentile at pre-test. As previous studies showed differences in P300 amplitude between low- and high-fit children [41,42], it is likely that aerobic and coordinative training did not lead to a change in this component due to a smaller adaptation reserve in already high-fit children. However, it should be noted that aerobic and coordinative training both increased aerobic fitness despite the high initial Shuttle Run performance. Consequently, these programs seem to be appropriate to promote children’s health by increased aerobic fitness rather than cognitive performance.

The present findings should not be interpreted without considering the study limitations. Despite the prescription of age-appropriate forms of training to AER and COR, the drop-out rate was relatively high (about 25%) in both groups. Although this resulted in a loss of statistical power, the magnitude of the effects sizes (η^2^ ≤ 0.045) indicate that this cannot explain the lack of significant time by group interactions for the primary outcomes. Furthermore, the training sessions in AER and COR were performed after school during leisure time. In contrast, meta-analytical findings suggest that training programs included in the school curriculum lead to greater benefits for inhibitory control [13]. Consequently, it cannot be ruled out that the prescribed training programs would be effective when implemented in another setting. Additionally, it is likely that a combination of the aerobic and coordinative training rather than one of these types alone would have elicited inhibitory control benefits similar to a previous study [19]. With regard to the intensity of the training programs, there was a higher heart rate in AER compared to COR. Thus, it cannot be ruled out that training programs matched for cardiovascular load, but with different coordinative demands would have resulted in a different pattern of results. Based on Norten et al. [43], training in AER and COR corresponded to moderate intensity and previously, benefits for inhibitory control were observed after an exercise program with similar intensity [16,19]. Other studies have found comparable improvements in the executive function domain after continuous training at higher intensity [15,18] or high-intensity interval training [44]. As evidence on the dose-response relationship is limited, it remains to be elucidated whether or not higher intensity promises greater benefits for inhibitory control. Moreover, the present findings may have been influenced by the choice of the activity in the control group. Although assisted homework sessions are not expected to improve inhibitory control, an effect cannot be ruled out. Based on criticism regarding the use of passive control groups in studies targeting cognitive function [33], this approach was chosen to reduce differences in expectations, social engagement and interaction with the supervisor between the intervention and control groups. Another limitation that may have confounded the present findings is the control for specific covariates. Inter-individual variance in pubertal status, which has an important role for the development of the brain [45], was reduced by including prepubescent children only. In contrast, no data was collected on the participants’ socioeconomic status and intelligence, although both variables are associated with executive functions [46,47]. However, it should be noted that evidence regarding the influence of both variables on training-induced benefits is lacking. In this respect, cross-sectional findings showed independent associations of physical fitness and socioeconomic status with executive function [48]. Consequently, it is unlikely that variations in socioeconomic status explain the absence of beneficial effects of aerobic and coordinative training on inhibitory control. Moreover, the comparability of the findings with previous studies investigating the effects of physical training on the P300 component is limited due to the use of different inhibitory control tasks [14,19,27]. Nonetheless, the modified Flanker task used in the present study induced the expected interference effect [23], which was evidenced by shorter P300 latency, reaction time, and higher accuracy on congruent compared to incongruent trials.

## 5. Conclusions

In the present study, no differences regarding the efficiency of aerobic and coordinative training for the enhancement of inhibitory control were found as both training programs failed to elicit changes in behavioral and neurocognitive measures. This finding also indicates that such training programs have no negative influence on the inhibitory aspect of executive function. However, aerobic and coordinative training led to an improvement in cardiovascular fitness, thus contributing to a domain also related to children’s health.

## Figures and Tables

**Table 1:** Participants’ characteristics.

	AER (*N* = 11)		COR (*N* = 12)		CON (*N* = 14)	
	M	SD	M	SD	M	SD
Age in y	9.1	0.6	9.6	0.8	9.3	0.6
Body mass index in kg/m^2^	16.2	4.4	17.5	3.2	16.9	1.5
Height in cm	138.5	18.5	140.8	10.2	139.7	5.5
Tanner score	1.0	0.0	1.2	0.4	1.4	0.7

Note: AER = aerobic training group; COR = coordinative training group; CON = control group.

**Table 2:** Behavioral performance on the Flanker task at pre- and post-test displayed for each group.

	Group	Pre		Post	
		M	SD	M	SD
Completed stages on Shuttle Run	AER	5.1	2.0	5.9	2.1
	COR	5.4	1.8	5.8	1.8
	CON	6.3	1.4	6.4	1.5
Total Score on HGMT	AER	530.2	50.3	560.6	55.7
	COR	571.1	29.2	597.0	38.2
	CON	563.9	53.0	573.1	44.4

Note: AER = aerobic training group; COR = coordinative training group; CON = control group; HGMT = Heidelberger Gross-Motor Test.

**Table 3:** Behavioral performance on the Flanker task at pre- and post-test displayed for each group.

	Trial Type	Group	Pre		Post	
			M	SD	M	SD
Reaction time in ms	Congruent	AER	747.3	141.0	749.6	114.1
		COR	713.5	69.4	714.9	75.4
		CON	654.3	159.8	676.4	134.7
	Incongruent	AER	778.1	138.9	808.9	129.8
		COR	749.3	82.7	763.4	90.0
		CON	698.0	177.1	726.4	154.4
Accuracy in %	Congruent	AER	70.8	17.9	78.3	11.6
		COR	78.3	13.3	86.1	10.0
		CON	71.0	11.6	79.5	11.1
	Incongruent	AER	63.2	18.0	72.9	13.4
		COR	71.5	13.4	82.9	9.7

Note: AER = aerobic training group; COR = coordinative training group; CON = control group.

## Supplementary Materials

The following are available online at http://www.mdpi.com/2077-0383/8/2/184/s1, Table S1: Example sessions for the aerobic training, Table S2: Example sessions for the coordinative training, Table S3: Results of the examination of gender-specific effects on the change in neurocognitive (P300 amplitude and latency) and behavioral measures of cognitive performance (reaction time and accuracy).
