# Supplementary material for: A Randomized Controlled Trial on the Effects of Aerobic and Coordinative Training on Neural Correlates of Inhibitory Control in Children
Source: J Clin Med. 2019 Feb 4;8(2):184. doi: 10.3390/jcm8020184 (PMC6406880; doi:10.3390/jcm8020184)
Supplement: Supplementary file 1 [file jcm-08-00184-s001.pdf]

Table 1. Example sessions for the aerobic training.

Example	Time	Exercise	Material	Remarks
1	10 min	Tail catching	Bands	Children have to steal each other's tails.not allowed to stand against the wall.
	10–15 min	Carp game	dice, staked running field	The children play in groups of two. Everyone has a dice. The number on the dice determines the number of laps the partner has to run.
	15–45 min	Game of rounders	ball, boxes, cones	Additional condition: children can be caught if standing still on one spot. They have to always move along the edge.
2	10 min	Keep the basket full	Balls and Box	A top part of the box is turned upside down and filled with gymnastic balls. A player 1 stand at the top of the box and throw out as many balls as possible from the box. The other players return the balls to the box as quickly as possible.<br>If the box is completely empty player 1 has won. .
	10-15 min	Formula 1	Boxes are positioned upside down on rolling boards.	Two children together, one sitting in the box the other one sliding the vehicle around the course.<br>After 3 laps, drivers change. Each child should drive 3 times. The first to get to the finish line wins.
	15–45 min	Construct-Deconstruct	Small boxes, ropes, medicine balls, etc.	Using different objects one big device is built by each team on one side of the field.<br>The task is to deconstruct the big device as quickly as possible and reconstruct it on the opposite site of the field. Each Team member can only carry one object at a time!

Table 2. Example sessions for the coordinative training.

Example	Time	Exercise	Material	Remarks
1	10 min	Tail catching	Bands	Additional task: Children who lost their tail, must stand on a Pad until they are set free by another child by means of running to laps around the trapped child. They are then allowed to grab a new band from the middle of the field very quickly making sure they are not caught. The one who collected the most tails wins.
	10–15 min	Hitting targets.	Balls, cones, box, target	Small groups collecting points. Boy and girls are evenly split into groups.<br>1. station: basketball pitch<br>2. station: tennis balls into the box<br>3. station: bowling<br>4.station: one child stands on the box with an cone upside down in its hand ready to catch. The other student throws the tennis ball into to cone.<br>Station 5: different target are marked on the wall and must be hit with the balls.
	15–45 min	Game of rounders with obstacles	Benches, Togupads, cones for slalom	Children stand on the Togupad or on a medicine ball balancing while playing rounders.
2	10 min	Warm up passing the ball	Balls	Two partners are moving through the hall passing the ball to each other.<br>Variant 1: After each pass turn around own body axis.<br>Variant 2: After each pass: squat jump
	10–15 min	Obstacle course (each station about. 4 min)	Cones, 2 Pedalos, coordination supervisor, Todupads, balls, ropes, boxes, balls	1. station: Pedalo-course. Cones mark course: drive through with the Pedalo.<br>2. station: coordination supervisor. design step and jumping sequence as a suggestion.<br>3. station: Togupads. Two opposite: passes to each other.<br>4. station: skipping. Different styles, forward backward, one-legged.<br>5. station: two children face each other on boxes. Pass balls in as many styles as possible. One-legged. Weak hand, from behind through the legs.
	15–45 min	Launch	Balls	Two partners stand behind each other at a distance of about 3 meters. The rear throws the ball over the partner’s head in a high arc allowing him to catch it after a single floor contact. After 2 to 5 minutes, partners change positions.<br>Important: it’s all about team work not how far the ball goes.

Table 3. Results of the examination of gender-specific effects on the change in neurocognitive (P300 amplitude and latency) and behavioral measures of cognitive performance (reaction time and accuracy).

	Interaction	df	Wilks' λ	F	p	η2
**P300**	Time × sex	2, 28	0.973	0.386	0.683	0.027
	Time × sex × group	4, 56	0.982	0.126	0.973	0.009
**Cognitive performance**	Time × sex	2, 30	0.907	1.539	0.231	0.093
	Time × sex × group	4, 60	0.917	0.668	0.617	0.043
